# Event Camera Simulator Improvements via Characterized Parameters

**DOI:** 10.3389/fnins.2021.702765

**Published:** 2021-07-27

**Authors:** Damien Joubert, Alexandre Marcireau, Nic Ralph, Andrew Jolley, André van Schaik, Gregory Cohen

**Affiliations:** International Centre for Neuromorphic Systems, The MARCS Institute for Brain, Behaviour, and Development, Western Sydney University, Kingswood, NSW, Australia

**Keywords:** event-based algorithms, event cameras, SNN benchmarks, simulator, SNN algorithm

## Abstract

It has been more than two decades since the first neuromorphic Dynamic Vision Sensor (DVS) sensor was invented, and many subsequent prototypes have been built with a wide spectrum of applications in mind. Competing against state-of-the-art neural networks in terms of accuracy is difficult, although there are clear opportunities to outperform conventional approaches in terms of power consumption and processing speed. As neuromorphic sensors generate sparse data at the focal plane itself, they are inherently energy-efficient, data-driven, and fast. In this work, we present an extended DVS pixel simulator for neuromorphic benchmarks which simplifies the latency and the noise models. In addition, to more closely model the behaviour of a real pixel, the readout circuitry is modelled, as this can strongly affect the time precision of events in complex scenes. Using a dynamic variant of the MNIST dataset as a benchmarking task, we use this simulator to explore how the latency of the sensor allows it to outperform conventional sensors in terms of sensing speed.

## 1. Introduction

There is a strong trend toward using simulation to increase the performance of machine vision algorithms and to explore their behaviour across a wide range of visual scenes and sensing scenarios. This allows for the exploration of critical situations, edge cases, and failure modes that would be impractical to test manually or collect via experimentation. This is often the case with real-world problems, where searching for every edge case is almost impossible. Even deep learning datasets, which have grown to be immense in size (Caesar et al., 2020), tend to only focus on well-defined sub-problems or sub-tasks, rather than providing a comprehensive dataset to understand the limitations and impacts of the sensor inside a final system. Tasks in which datasets are difficult to obtain commonly arise in applications of neuromorphic technologies. Take the example of neuromorphic vision sensors applied to tracking objects in space through telescopes for the purpose of predicting collisions (Cohen et al., 2019; Zołnowski et al., 2019). It is difficult to assess the performance of such a system (Afshar et al., 2019) as the collision between satellites cannot be emulated in the real-world using fake targets, as is commonly done in validating automotive applications. In this application, the diversity of the high-speed objects passing through the field of view requires sensors that are fast and do not have predetermined acquisition rates. As with many real-world applications, ground-truth for space object tracking is difficult, if not impossible, to acquire. Even when there is candidate ground truth available, there may be unknown elements in the data, such as a small and previously undetected piece of debris in the field of view that was not detectable by the sensor producing the ground-truth data. Assessing a new sensor with a novel means of signal acquisition against a fundamentally different sensor cannot be expected to produce reliable or validated ground truth.

Simulation is an important step in the design and development of novel sensors. The development of new neuromorphic sensors requires a robust simulation platform, a well-established method of validating the simulator, and a means to produce meaningful measurements beyond simply assessing signal-to-noise ratio. The use of simulators to uncover performance optimisations will allow for more rapid development of application-specific sensor designs, without the high costs and barriers-to-entry involved in silicon device design and fabrication. The solution to this lack of data is not simply to collect more. This is especially relevant when exploring novel sensor types, as new data would need to be collected for every iteration of the sensor design, making the validation of such datasets unsustainable. It is possible to augment the lack of data through the use of simulation. However, this is subject to how well the model corresponds to reality, and simulated systems often pose far simpler problems than their real-world counterparts (Jakobi et al., 1995). For visual sensors, the simulation is often not restricted to just the visual scene but requires a detailed model of the sensing hardware itself. For novel sensors, this requires new models that need to be created and validated. When designing novel sensors, the transfer function implemented in the model of the sensor can be fundamentally different, and this can have a significant impact on the accuracy of the simulator.

**Figure 1:**
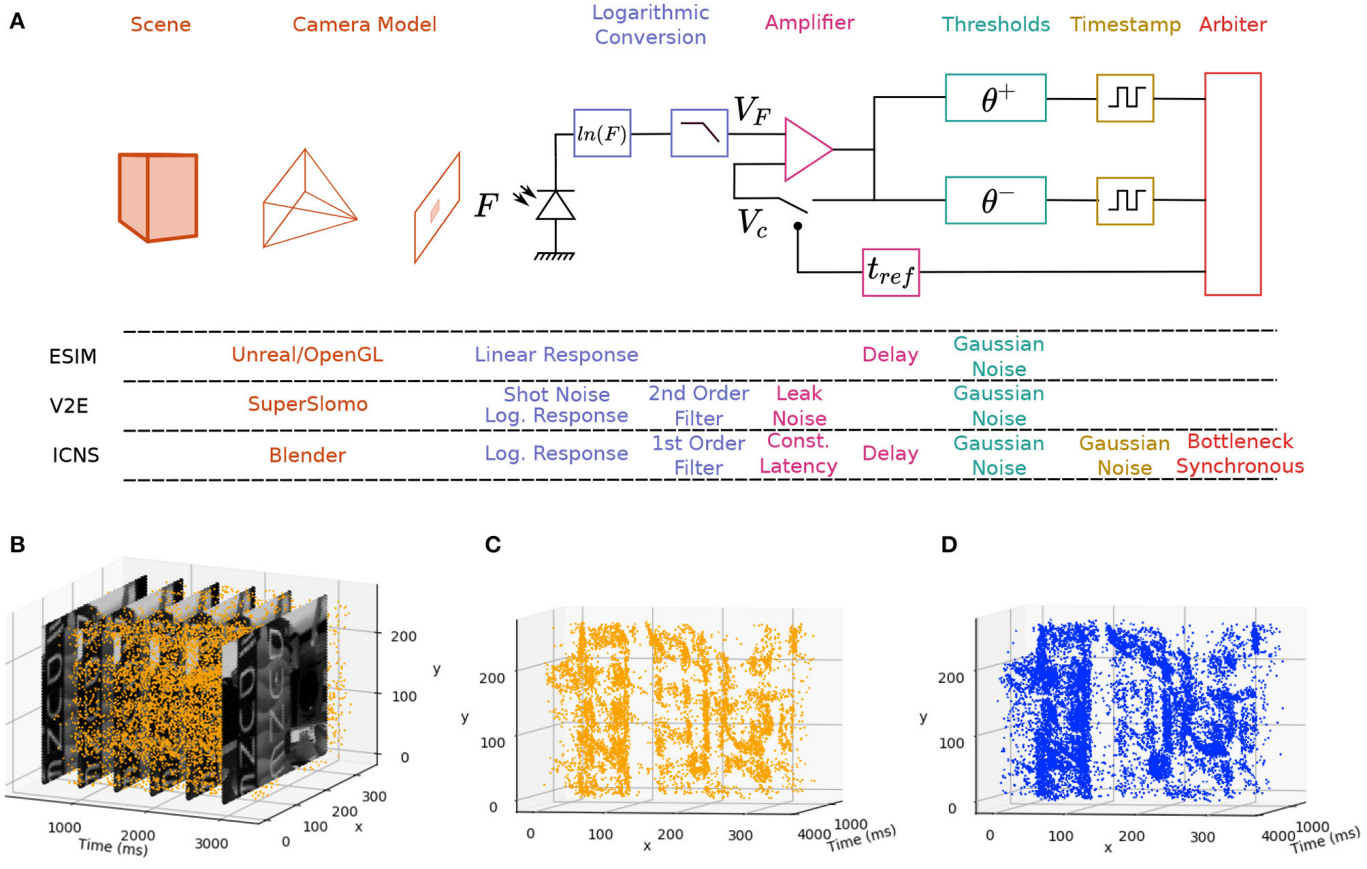
**(A)** Main blocks of an imaging system based on a DVS camera simulated by ESIM, V2E, and our model (ICNS). The Scene and Camera Model are often part of the same rendering tool, but slow motion interpolation neural networks such as SuperSlomo can help provide better time precision. The Logarithmic Conversion block encapsulates the conversion of the light into an electrical signal before the Amplifier and the Thresholds. In the ICNS model, the noise is also injected before the Timestamp block and the noise distribution is a function of the light level. **(B)** Events and frames from the DAVIS and comparison between the original events **(C)** and the simulated events **(D)** using the ICNS model.

This paper examines the state of various DVS simulators, and proposes a new simulation model that better reflects the true behaviour of these devices. This model is then validated against a real device, giving credence to both the model and the simulator. This simulation platform can then be used to explore different designs for neuromorphic vision pixels by allowing for the simulation and the comparison of the results when applied to dynamic tasks. A summary of the different contributions of the simulator is presented at Figure 1.

## 2. Simulating an Event-Based Neuromorphic Pixel

This paper focuses primarily on neuromorphic vision sensors that emit data in an event-based rather than uniformly-sampled manner. Taking inspiration from biology, the field of neuromorphic sensing seeks to develop physical devices capable of matching biological organisms' abilities to sense and adapt to new environments. An important step to achieving this goal arose with the development of event cameras, such as the DVS (Lichtsteiner et al., 2008) and other neuromorphic sensors such as the ATIS (Posch et al., 2011) and the sensitive DVS (Serrano-Gotarredona and Linares-Barranco, 2013). These novel devices brought a new perspective to conventional approaches to vision and sensing through their sparse and event-based nature and activity-driven data output. These compelling features promised to change conventional computer vision's fundamental limits by allowing sparse, high-speed, and low-power visual sensing.

Event cameras have been applied in many diverse research fields where conventional algorithms traditionally struggle. Examples include applications in autonomous driving systems (Maqueda et al., 2018), high-speed trackers (Delbruck and Lang, 2013), and spatiotemporal pattern recognition tasks (Amir et al., 2017). A very comprehensive list of applications and algorithms can be found in Gallego et al. (2019). Whilst these applications leverage the benefits of the DVS data and have produced many promising proofs-of-concept, it has proved extremely difficult to translate these into reliable and computationally efficient algorithms suitable for real-world applications. Given the similar underlying concepts and inherently compatible approach to data, the processing of event-based sensors can be achieved using Spiking Neural Networks (SNN). Such networks now extend traditional conventional deep-learning architectures and implement them with local computing and learning rules. Even if these algorithms can reach similar accuracy (Rückauer et al., 2019), they still lag behind conventional neural networks in terms of over-fitting (the temporal information is normalised in the database) and inference time. Applications of DVS sensors often focus on the processing of recorded data, rather than matching the performance and requirements of the sensor to the specific intended application. This is in stark contrast to biology, where the sensor and processing are specialised for the required tasks.

A primary goal of this work is to explore how best to simulate event-based sensors, and how best to use such simulations to improve the performance of neuromorphic systems.

### 2.1. Event Pixel Models

The pixel design found in the DVS sensor was developed in the 1990s (Delbrück and Mead, 1989), arising out of an effort to mimic the behaviours of the ON/OFF cells in the retina. Each pixel implements some local processing, drawing on how information acquired by the cones and rods in biological retinas pass through many specific analogue computations before reaching the cortex (Masland, 2012; Seung and Sümbül, 2014). However, to keep the pixel size small, only a few operations are possible within each pixel on the focal plane. Devices with more complex and in-pixel processing do exist (Carey et al., 2013; Millet et al., 2018), but are not used as much as the simple pixel models. The first prototype event camera that saw widespread use was the DVS128 sensor (Lichtsteiner et al., 2008), enabled by its consistency and reliability. Industrialised upgrades (Son et al., 2017; Finateu et al., 2020) and miniaturised variants (SEES1 camera; Falanga et al., 2020) have since followed it. Our work examines the pixel variant found in these camera designs, which is built around three consecutive processing steps. Firstly, the photo-current *F* is logarithmically converted into a voltage *V*_*F*_, using the response of a transistor kept under its threshold with a feedback loop. In the second step, the temporal difference between *V*_*F*_ and the last voltage before the pixel was reset *V*_*c*_ is amplified using a capacitive amplifier. Finally, the output step checks if the amplified difference has crossed an externally-controllable positive or a negative threshold. If so, the pixel emits an event to indicate the change and the pixel reference voltage is reset (usually after a short refractory period). These cameras have a single output bus that implements the Address Event Representation (AER) protocol (Boahen, 2000), and an arbiter required to collate the events from the asynchronous pixels and mitigate collisions when events occur at the same time. The original asynchronous arbiters process row requests first and then column requests, adding an extra delay before the reset of the pixel. This directly modifies the transfer function of the pixel in a non-trivial manner. In fact, other readout strategies do exist and have their own characteristics, and the synchronous scanning of the pixel array found in Li et al. (2019) is a good example where the transfer function of the arbiter would be fundamentally different from the original analogue and asynchronous prototypes.

### 2.2. Existing DVS Simulators

The lack of easily available datasets has led to the use of pixel-level event-based simulators in several applications (Rebecq et al., 2018). These include the simulation of complex environments where the control strategy is in the loop (Kaiser et al., 2016) and the simulation of colour event-based sensors which are not yet widely available (García et al., 2016; Scheerlinck et al., 2019). The accuracy of these simulators varies from precise electrical simulations of the pixel (Remy, 2019) designed to optimise the sensor's performance, to high-level simulations of well-defined problems like the simulation of a moving bar (Barbier et al., 2020). These high-level simulations are often sufficient for understanding and exploring the performance of algorithms, and one of the first simulations of an event-based pixel was implemented to train a robot to follow a line (Kaiser et al., 2016). This is a good use of simulation as the robot will likely crash several times during learning, which is significantly less costly in simulation than in practice. In the model proposed in Kaiser et al. (2016), the pixel is simulated as a fixed delta modulator and *F*(*t*), the temporal light flux received by the pixel, is approximated by rendering a frame at every time step (*dt*). Depending on the quality of the rendering engine, the simulated flux has different ranges, precision, or units. In our model, the flux is scaled with the maximal value of the rendering engine. Because of this scaling, *F* does not have a unit. The response of the pixel is linear, and the voltage of the photosensitive frontend *V*_*F*_ equals *F*(*t*). The pixel has one internal state, *V*_*c*_(*t*), which is the voltage compared to thresholds to generate events and both flux and voltage values are normalised by the maximum value and are without any specific unit. If θ^+^ and θ^−^ are the positive and negative thresholds respectively, then the condition to create an event can be defined as:

(1) $$\begin{array}{l} V_{F}\left(t+dt\right)-V_{c}\left(t\right)>\theta^{+}\vee V_{F}\left(t+dt\right)-V_{c}\left(t\right)<\theta^{-} \end{array}$$

In this case, *V*_*c*_(*t* + *dt*) = *V*_*F*_(*t* + *dt*), and the timestamp of the related event is *t*_*ev*_ = *t* + *dt*.

Whilst being computationally fast, this approach has two main disadvantages. Firstly, the event created has the same timing precision as the simulated frames, which does not replicate the high temporal resolution of event-based sensors. Secondly, the logarithmic response of the sensor is also neglected in this model. This is not an issue in their case as the environment is a highly-controlled artificial scene.

The ESIM simulator (Rebecq et al., 2018) improves on this model by addressing these issues and adding extra features. The flux, converted to its logarithmic value, *V*_*F*_(*t*) = *ln*(*F*(*t*) + ϵ), is linearly interpolated between *t* and *t* + *dt* to determine when the threshold is crossed, as shown in the following equation:

(2) $$\begin{array}{l} t_{ev}=t+dt\frac{\theta -V_{F}\left(t\right)}{V_{F}\left(t+dt\right)-V_{F}\left(t\right)} \end{array}$$

The pixel is then quicker to react when the amplitude of the contrast increases, which is similar to the response of a real DVS pixel (Joubert et al., 2019). Interpolating the light flux between *F*(*t*) and *F*(*t* + *dt*) also allows for the creation of intermediate events during *dt* if the contrast change is large enough. However, in the ESIM model, an infinite contrast could generate an event instantly. That is not the case in physical devices as the following stage still slows down the pixel (Posch and Matolin, 2011) when the time constant of the logarithmic block is negligible. The latency is also a function of *dt*, which does not depend on the sensor itself but rather on the rendering frame rate.

In the same spirit as our work, V2E sought to improve the ESIM model and also provides several useful additions, including the ability to optimise the transfer function to match the behavior of a real sensor. In their model, the light level is converted into a current following a logarithmic response, which is linearised at the first order for low values, as *ln*[1 + *F*(*t*)] ~ *F*(*t*) when *F*(*t*) is small. This reduces the noise when the value of *F*(*t*) is small, since the pixel is less sensitive as *F*(*t*) ≤ *ln*[1 + *F*(*t*)] for positive *F*(*t*). But more relevant to our model, their simulator approximates the logarithmic conversion by simulating a discrete second order low pass filter, whose time constant τ is updated for every new amplitude of *F*(*t*):

(3) $$\begin{array}{l} \tau =\tau_{max}\frac{275}{F\left(t\right)+20} \end{array}$$

where *F*(*t*) ∈ [0, 255] and τ_*max*_ is the maximum time constant. This follows observations that the initial portion of the DVS pixel acts as a low pass filter whose time constant is inversely proportional to the ambient light level (Delbruck and Mead, 1994).

Taking noise into account in a simulation is a particularly difficult task. This is an important consideration as DVS pixel studies have simulated the noise of the threshold (Lichtsteiner et al., 2008; Nozaki and Delbruck, 2017), modelled here as Gaussian noise centred around the mean threshold: θ ~ *N*(μ(θ), σ(θ)). The mismatch of the threshold has been simulated, as in ESIM, but the negative or almost null thresholds are rejected. Indeed, a pixel with a very small threshold would be overly sensitive and therefore continually generate events, similar to what happens with a hot pixel in a physical camera. The noise model also simulates the leak effect of the amplifier reset transistor, only depending on the temperature (Nozaki and Delbruck, 2017), as well as the temporal noise simulated by a Poisson law whose rate decreases in low intensities to mimic the shot noise. The authors of Hu et al. (2021) also optimise the thresholds of the pixel to generate as many events as a real DVS camera would generate.

Moreover, V2E and ESIM use different approaches to decrease the computation cost. ESIM tackles the issue of the optimal *dt*. As it is computationally expensive to render the scene every microsecond, an estimation of the motion in the focal plane is used to find the optimal *dt*. If *v*_*max*_ is the maximum speed in the focal plane expressed in pixel per second (*px*.*s*^−1^), then the quickest object will move to the next pixel after $dt=v_{max}^{-1}$. This approach relies on a perfect estimation of the speed in the focal plane, which is precise as long as the optics model is accurate. V2E introduces a slow-motion network interpolating *F*(*t*) to obtain higher rendering rates. This network is trained using conventional frames and fails on scenes not suited for standard cameras, for example on which fast objects create motion blur. Thus, simulating events upon these interpolated values created events uncorrelated with the object. Rather than providing simulated frames to the pixel model, a video stream acquired with a conventional sensor would also provide similar input data (Gehrig et al., 2020). In this approach, a neural network is trained to convert the video stream into events. However, temporal artifacts created by the Image Signal Processor (ISP), for example from the auto-exposure algorithm, can cause more information to be lost. This issue can be compensated by increasing the frame rate of the video, but this can lead to additional and potentially unnecessary computations.

Our model builds upon the groundwork laid by these simulators, focusing on simulating noise and better estimations of the latency by adding the effects of the arbiter.

### 2.3. Improving on the DVS Camera Model

**Figure 2:**
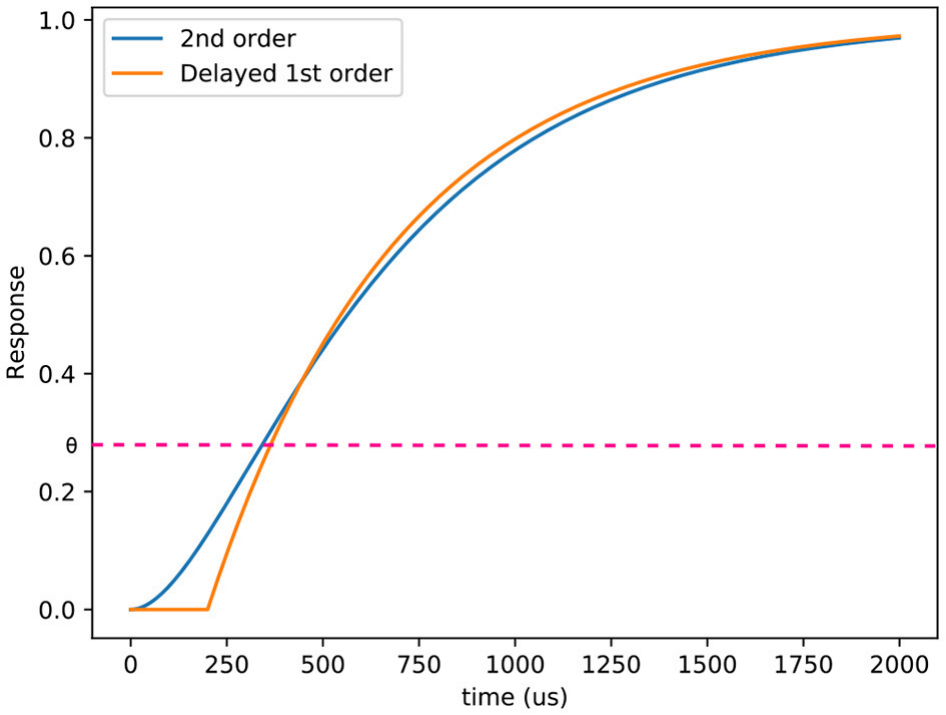
Comparison between the responses of a delayed first order low pass filter and a second order low pass filter. θ represents one of the thresholds of the comparators. In this case, τ_*c*_ = 200μ*s* and τ_*l*_ = 500μ*s*.

Our model of the DVS camera adds a number of improvements and additions to existing models. Existing pixel models do not incorporate many real-world phenomena, despite early experiments with the DVS pixel showing that its behaviour is affected by the ambient light (Lichtsteiner et al., 2008), the amplitude of the change, and the temperature of the sensor (Nozaki and Delbruck, 2017). A comparison between ESIM and our approach is provided Figure 2 to illustrate how the light is interpolated between two flux values. In our model, the temperature effects are neglected, and the model is only a function of the light level. The C++ and Python implementations are provided^1^.

#### 2.3.1. Latency and Sensitivity

Previous works (Lichtsteiner et al., 2008; Posch et al., 2011) mention that the latency of the DVS pixel follows a second order low pass filter with two different real poles. Let τ_*l*_ and τ_*c*_ be the time constants of the logarithmic conversion and the amplifier and comparator blocks. In this case, the transfer function *H* of the pixel before conversion to events can be modelled as follows:

(4) $$\begin{array}{l} H\left(s\right)=\frac{1}{\left(1+s\tau_{l}\right)\left(1+s\tau_{c}\right)} \end{array}$$

Where *s* is the complex frequency of the stimulus. The time constant of the logarithmic conversion of the pixel is inversely proportional to the photocurrent, which implies that one pole of the second order filter changes in the focal plane. The temporal response of the pixel follows the equation:

(5) $$\begin{array}{l} V_{c}\left(t+dt\right)=\left(V_{F}\left(t+dt\right)-V_{c}\left(t\right)\right)\left(1-\frac{\tau_{c}e^{-\frac{dt}{\tau_{c}}}-\tau_{l}e^{-\frac{dt}{\tau_{l}}}}{\tau_{c}-\tau_{l}}\right) \end{array}$$

**Figure 3:**
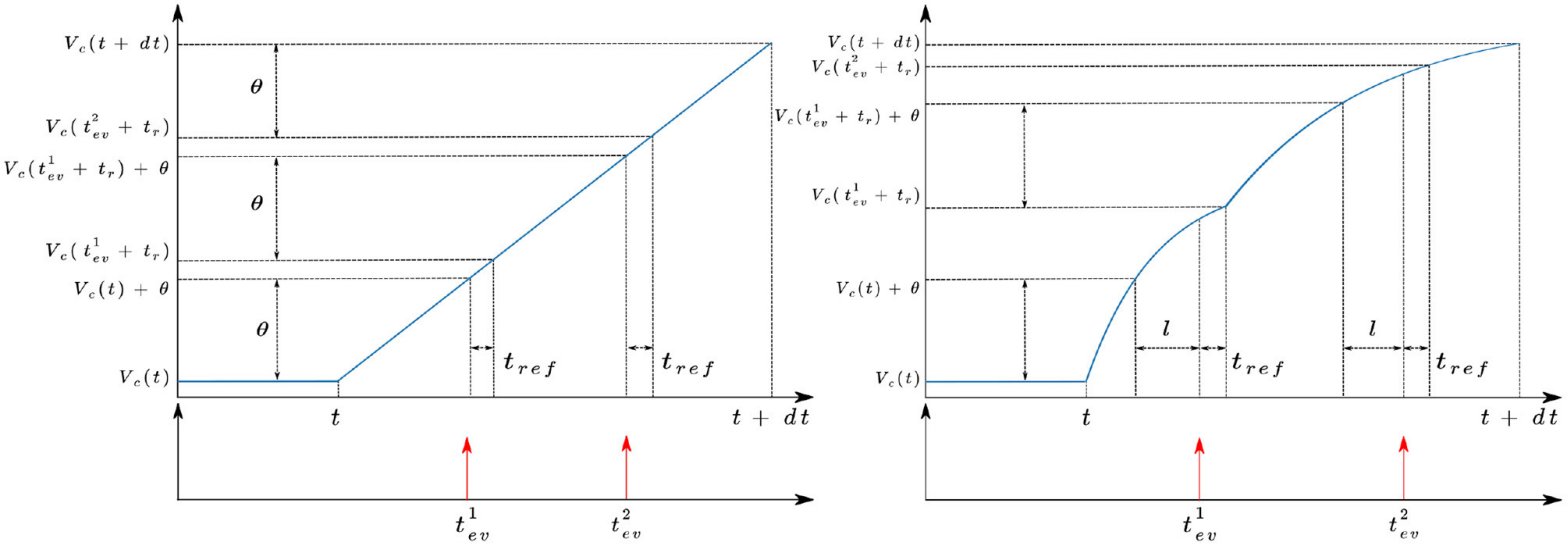
Behavior of the ESIM **(left)** and ICNS **(right)** models between two simulated images at time *t* and *t* + *dt*.

Given a temporal relative contrast of the light received by the pixel and a threshold, this equation has no analytical solution to find the corresponding timestamp. To help solve this equation, as τ_*l*_ depends on the light level (Delbruck and Mead, 1994; Hu et al., 2021), we consider that the mean latency of the next stage, *l*, is independent from the lighting conditions. An example of a delayed first order low pass filter and a second order low pass filter is presented at Figure 3 and illustrates that the behavior of the pixels is mainly different under the threshold. This approximation diverges from the behavior of the pixel, but allows it to simulate it's response if *dt* varies. Since the rendering rate must be adapted to the dynamics of the scene, this feature is essential.

Only the logarithmic conversion behaves as a low pass filter, and the delay to cross the threshold of the comparator is expressed as:

(6) $$\begin{array}{l} t_{ev}\approx t+l-\tau_{l}(t)ln(1-\frac{\theta }{V_{F}\left(t+dt\right)-V_{c}\left(t\right)}) \end{array}$$

The time constant τ_*l*_, is inversely proportional to the amplitude of the change when the latter is high, and if *F*_*m*_ is the maximum flux that the pixel can receive, then $\tau_{l}\left(t\right)=\tau_{l}\frac{F_{m}}{F\left(t\right)}$. When an event is generated, the final full expression of the timestamp is given by:

(7) $$\begin{array}{l} t_{ev}\approx t+l-\tau_{l}\frac{F_{m}}{F\left(t\right)}ln(1-\frac{\theta }{V_{F}\left(t+dt\right)-V_{c}\left(t\right)}) \end{array}$$

If no event is generated, the response behaves as a delayed first order low pass filter. The expression of the latency in Equation (7) depends on the threshold of the sensor, which is normally distributed. If *j* is the standard deviation of *l*, the final standard deviation of the latency is given by propagating the error into Equation (7):

(8) $$\begin{array}{l} \sigma \left(t_{ev}\right)=\sqrt{j^{2}+{\left(\tau_{l}\left(t\right)\frac{F_{m}}{F\left(t\right)}\frac{\sigma \left(\theta \right)}{V_{F}\left(t+dt\right)-V_{c}\left(t\right)}\right)}^{2}} \end{array}$$

In ESIM, the refractory period of the sensor, *t*_*ref*_, is modelled as a simple condition which prevents a pixel from firing if the time difference between an event and the last one is too short. In our model, the potential of the pixel is updated at *t* + *t*_*ev*_ + *t*_*ref*_ and not at *t* + *t*_*ev*_ like ESIM does. Moreover, if *t*_*ev*_ + *t*_*ref*_ > *dt*, then the potential is updated for the next simulated image.

**Figure 4:**
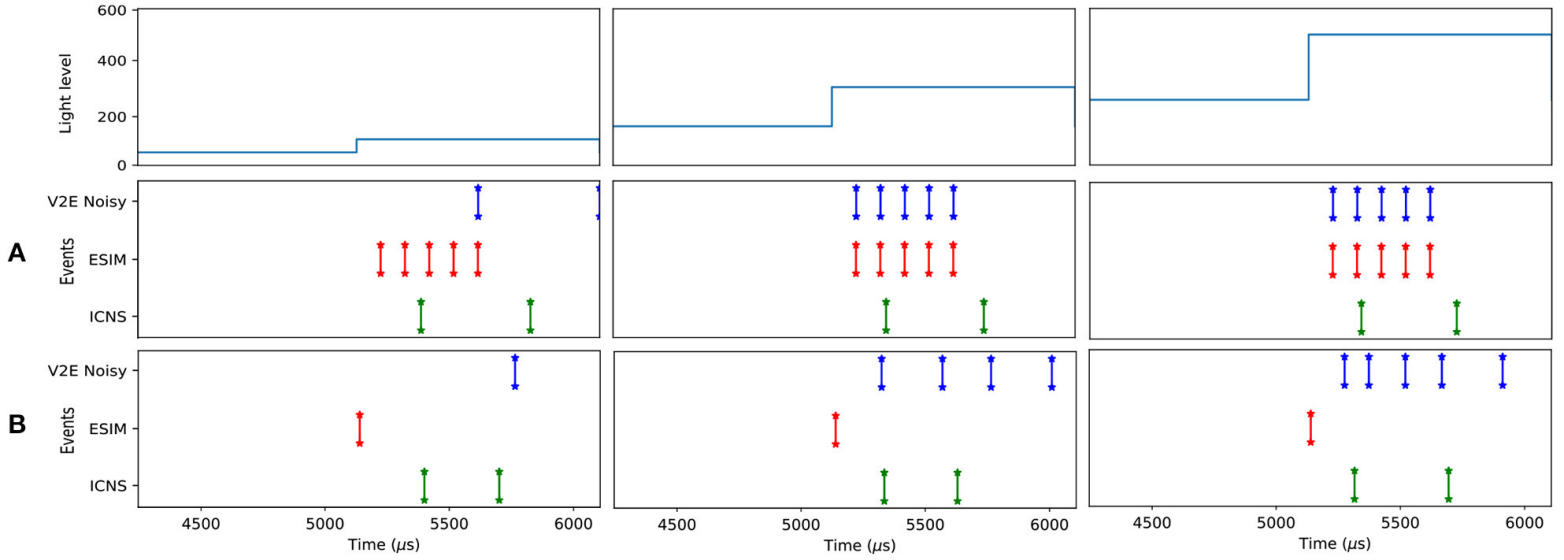
Comparison of the response of the ESIM, ICNS, and V2E pixel when the light changes with a constant relative contrast equal to 200%, but different background light levels (50, 150, and 250). The contrast threshold is set to 0.15 (deviation 0.02). The temporal characteristics of the V2E pixel follows the noisy parameters (https://github.com/SensorsINI/v2e), except for the cutout frequency equals to 300 Hz. The parameters of the ICNS model's latency are *l* = 100μ*s* and τ_*l*_ = 500μ*s*. For **(A)**, *dt* = 500μ*s*, and **(B)**, *dt* = 50μ*s*.

A comparison between the different models is presented in Figure 4 for a positive contrast with different light levels. These illustrations demonstrate how our model does not produce events proportional to contrast (as with ESIM), nor is it affected by the rendering rate (as in V2E). Even though the delayed first order low pass filter is an approximation, it allows to render the scene using a variable rendering rate to save unnecessary computations, while preserving the fidelity of the event timestamps.

**Figure 5:**
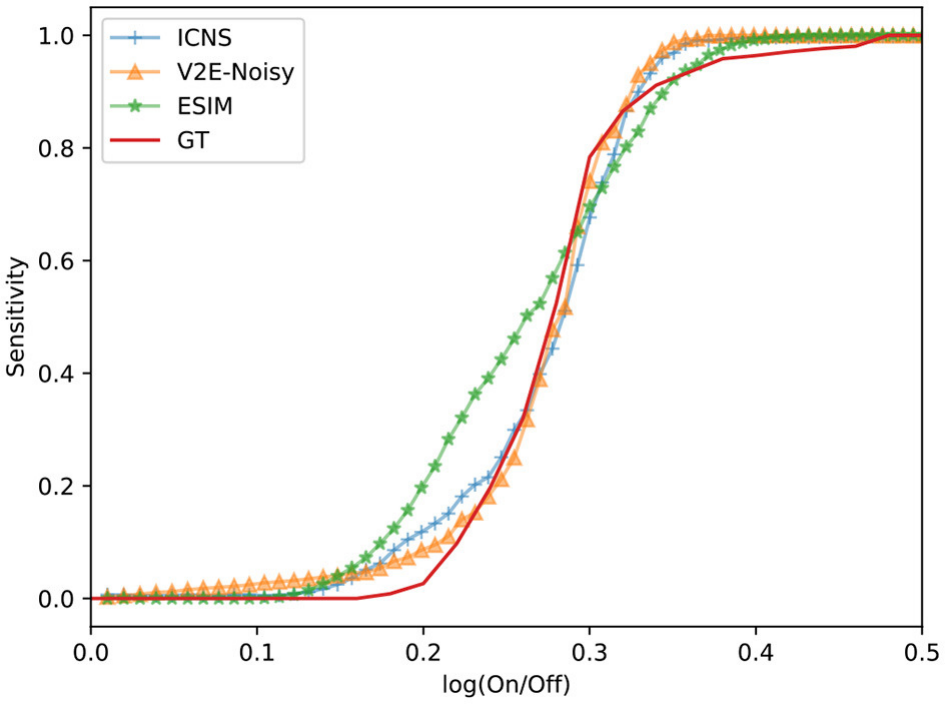
Sensitivity of the different models as a function of the relative contrast $\frac{On}{Off}$. The sensitivity measured on a real DVS pixel is also provided (GT). Both real and simulated pixels are stimulated with a square wave (10 Hz frequency), whose amplitude increases monotonically from 0 to 0.5 log. units.

The latency of the pixel is highly related to the sensitivity and can be characterised with its cumulative probability to generate an event as a function of the contrast (Posch and Matolin, 2011). The experiment consists of presenting the same contrast several times and computing the probability that the pixel reacts before the next change. This curve is measured for both models on Figure 5 using two different sampling rates (1 and 0.1 ms). The sensitivity of the different models does not depend on the rendering rate because the frequency of the stimulus is chosen to be high enough to neglect time constants. When a periodic stimulus has a low contrast close to the threshold of the pixel, after detecting the change, the pixel can be reset in a state where the new relative contrast is now bigger than the threshold. In this case, the pixel is stuck and cannot detect new periods of the signal. The theoretical S-curve equation *S* can be approximated as:

(9) $$\begin{array}{l} S\left(c\right)=\frac{1}{n}\underset{i\in \left[1..n\right]}{\sum }P{\left(2\theta >ln\left(c\right)\right)}^{2}\\ \;=\frac{1}{n}\underset{i\in \left[1..n\right]}{\sum }(\int_{-\infty }^{ln\left(c\right)}\frac{1}{2\sigma \left(\theta \right)\sqrt{\pi }}e^{-{\left(\frac{\theta -2\mu \left(\theta \right)}{\sqrt{2}\sigma \left(\theta \right)}\right)}^{2}}d\theta {)}^{2} \end{array}$$

Where *n* is the number of periods and *c* the contrast. In this case, the sensitivity of the pixel is fixed to 0.15, which corresponds approximately to half the value of the point where the cumulative distribution function reaches 0.5.

In recent years, the design of the DVS pixel has not changed substantially, but the arbiter has been significantly improved. For example, the work of Son et al. (2017) groups the pixels in blocks before the arbiter, while Li et al. (2019) uses a synchronous readout. Our simulator aims at simulating different arbiters as they can directly affect the timestamping accuracy of the events.

#### 2.3.2. Arbiter

The algorithms presented in this section are detailed in the Appendix. This work first introduces a simplified behaviour of the AER arbiter in which the model assumes that a given number of events can be processed between two simulated frames, and can be thought of as a bottleneck accumulator with a fixed bandwidth.

Increasing the number of pixels simulated per time step increases the latency added by the arbiter (Joubert et al., 2019). With *n*_*u*_(*t*) representing the number of events accumulated before the arbiter at the time *t*, the latency of the arbiter is given by the relation *l*_*AER*_(*t*) = *n*_*u*_(*t*)*l*_*AER*_(0) where *l*_*AER*_(0) is the latency of the arbiter to process one event. The maximum number of events processed between the last two simulated frames is equal to *n*_*p*_(*t*) = *dt*/*l*_*AER*_(*t*). The timestamp of every event produced by the filter is increased by *l*_*AER*_(*t*). Since the pixels are reset before the arbiter, the refractory period does not include the contribution of the arbiter. Thus, two events at the same location cannot be accumulated in the arbiter and the latest event will be discarded. This implementation of the bottleneck arbiter is fully described in the Appendix section (Algorithm 3), and can be applied to the events generated by the V2E, ESIM (Algorithm 1) or ICNS (Algorithm 2) model of the pixel.

**Figure 6:**
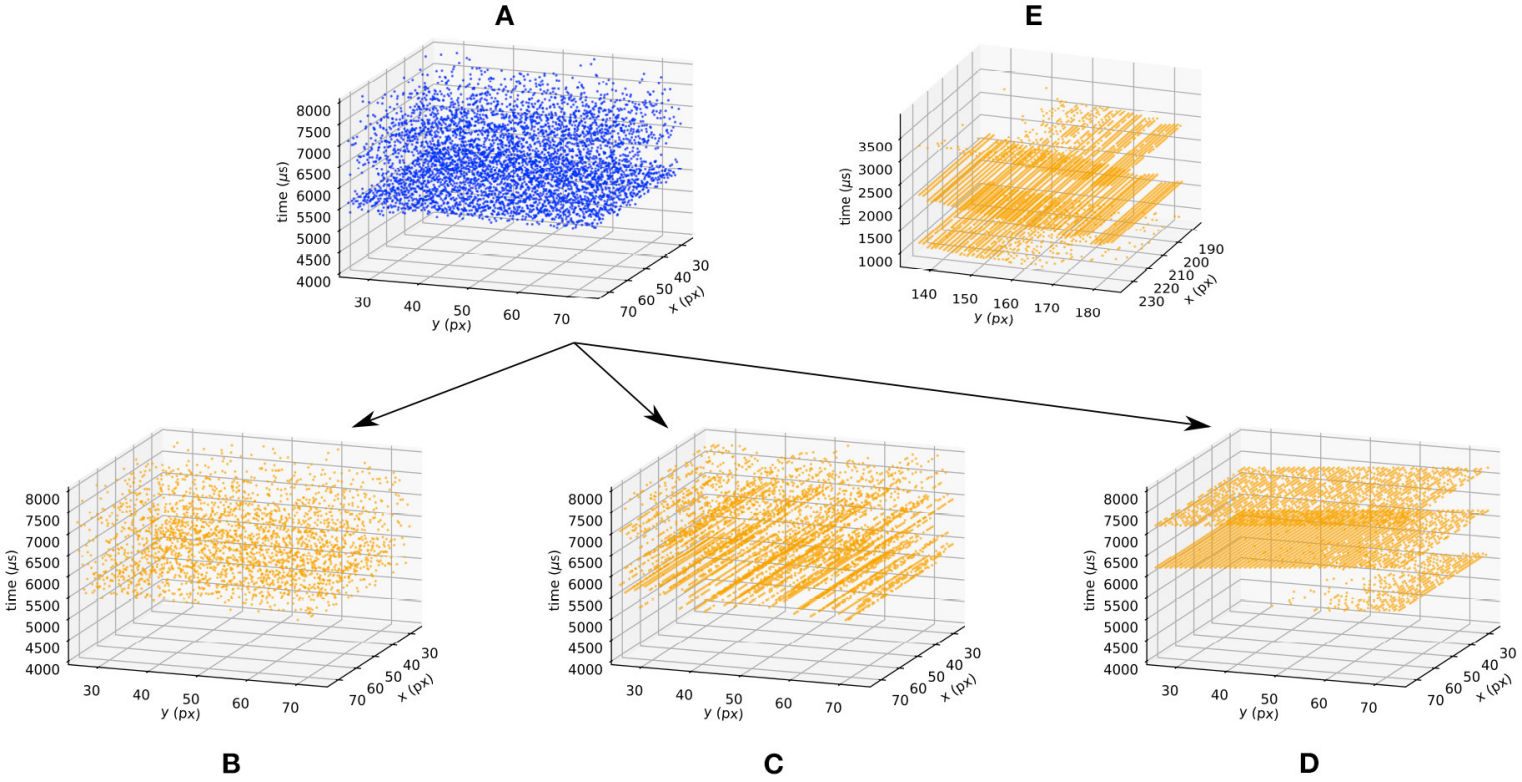
Effect of the arbiter when the background light is flashing. Blue (orange) events correspond to a sensor without (with) an arbiter. The blue events **(A)** are generated using the ICNS model when the light is turned on at *t* = 5, 000. The bottleneck arbiter **(B)**, asynchronous **(C)**, and synchronous **(D)** row models are applied to the blue events. The data provided by real DVS pixels are provided **(E)** as a matter of comparison.

This asynchronous row algorithm is described in Algorithm (4). The recent DVS prototypes also feature a synchronous readout of the events (Li et al., 2019; Suh et al., 2020). In this case, rather than randomly selecting one row, they are scanned at a given rate *f*_*r*_. This implementation is described in Algorithm (5) and the different methods are compared to the events generated by a real DVS imager in Figure 6. To test this implementation, we use a flashing light to trigger all the pixels at the same time. For both architectures processing row by row, artificial horizontal rows of events are observed in real DVS when the scene is not sparse (Suh et al., 2020). In Figure 6, the real DV346 sensor used as a matter of comparison behaves as a synchronous row arbiter. Using an ATIS, the arbiter behaves as an asynchronous row readout. Thus, the model of the arbiter chosen depends on the final sensor used to tackle a given application.

#### 2.3.3. Temporal Noise

The noise of the reset amplifier at the comparator stage is modelled through the distribution of the thresholds. However, the sensor still generates background events dependent on the irradiance level (Moeys et al., 2018) and the temperature (Nozaki and Delbruck, 2017). This extra noise cannot be solely linked to the reset noise, because even if its effect is amplified for high irradiances, other noise sources like dark current and shot noise create imperfections. In our model, this noise is only characterised through its cumulative distribution function. The principal limitation of such an approach lies in the variations of this distribution when the order of magnitude of the background light level changes. In this case, the distribution is shifted in the frequency domain.

To provide a generic approach, each pixel has a unique noise distribution which is shifted as soon as the order of magnitude of the light level changes. By providing the next noise event timestamp to the block simulating the temporal noise, we avoid generating a random variable at each timestep. At the beginning, each pixel owns a random phase ϕ selected following a uniform law on the interval [0, 1/μ(*BGN*)], with μ(*BGN*) being the mean frequency of the noise, often referenced as the background noise of the sensor. Then, when *t* > ϕ, a noise event is generated. To estimate the time interval *t*_*noise*_ of the next noisy event, the cumulative distribution function is used. If *P*(*dt*_*noise*_) is the probability of generating a noise event in the next *dt*_*noise*_ μs, then:

(10) $$\begin{array}{l} f\left(dt_{noise}\right)=\int_{-\infty }^{dt_{noise}}P\left(x\right)dx \end{array}$$

**Figure 7:**
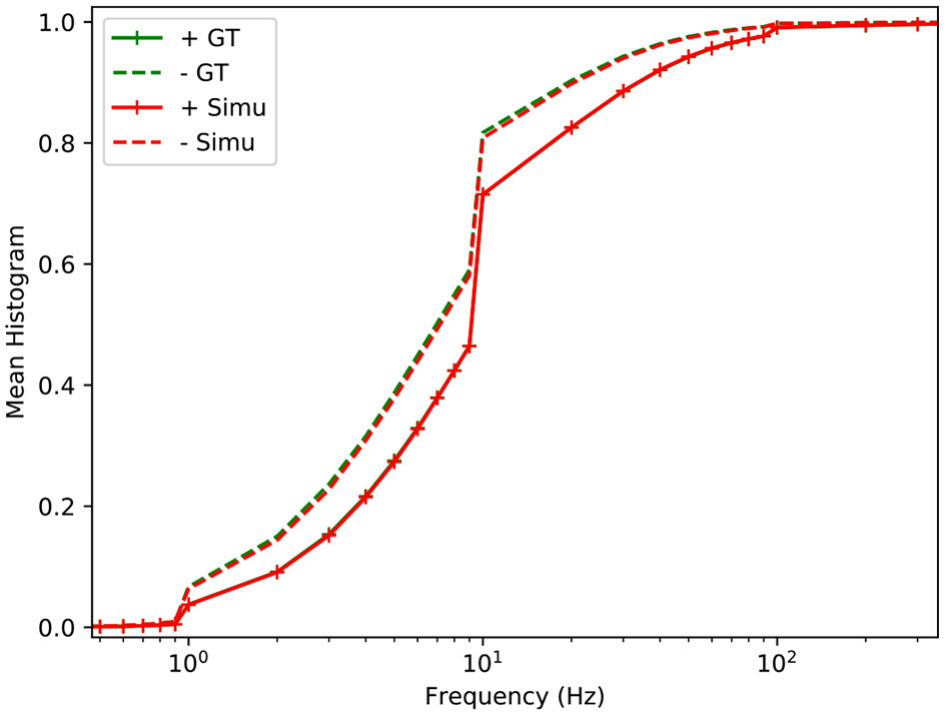
Cumulative distribution function of the relative spectrum of the ATIS (Posch et al., 2011) positive and negative background noise (irradiance 0.1 lux), for real data (green) and simulated (red).

By randomly selecting a value α between 0 and 1, *t*_*noise*_ can be estimated as follows: $t_{noise}=f^{-1}\left(\alpha \right)$. Figure 7 shows the distributions of the noise for a real and a simulated pixel whose light level is constant. The observed noise spectra are similar.

The next section illustrates how the simulation model can be used, and how it could help to understand the link between the trajectory of the eye, its impact on the events generated and the performance of a classifier.

## 3. Experiments

The first experiment in this section compares the different models for an office scene, illustrating the difference between the model's output and the output of a real sensor. The second experiment shows how the whole pipeline makes better use of the time information encoded with the events by simulating a reading task based on a classification dataset.

### 3.1. Office Scenes

**Figure 8:**
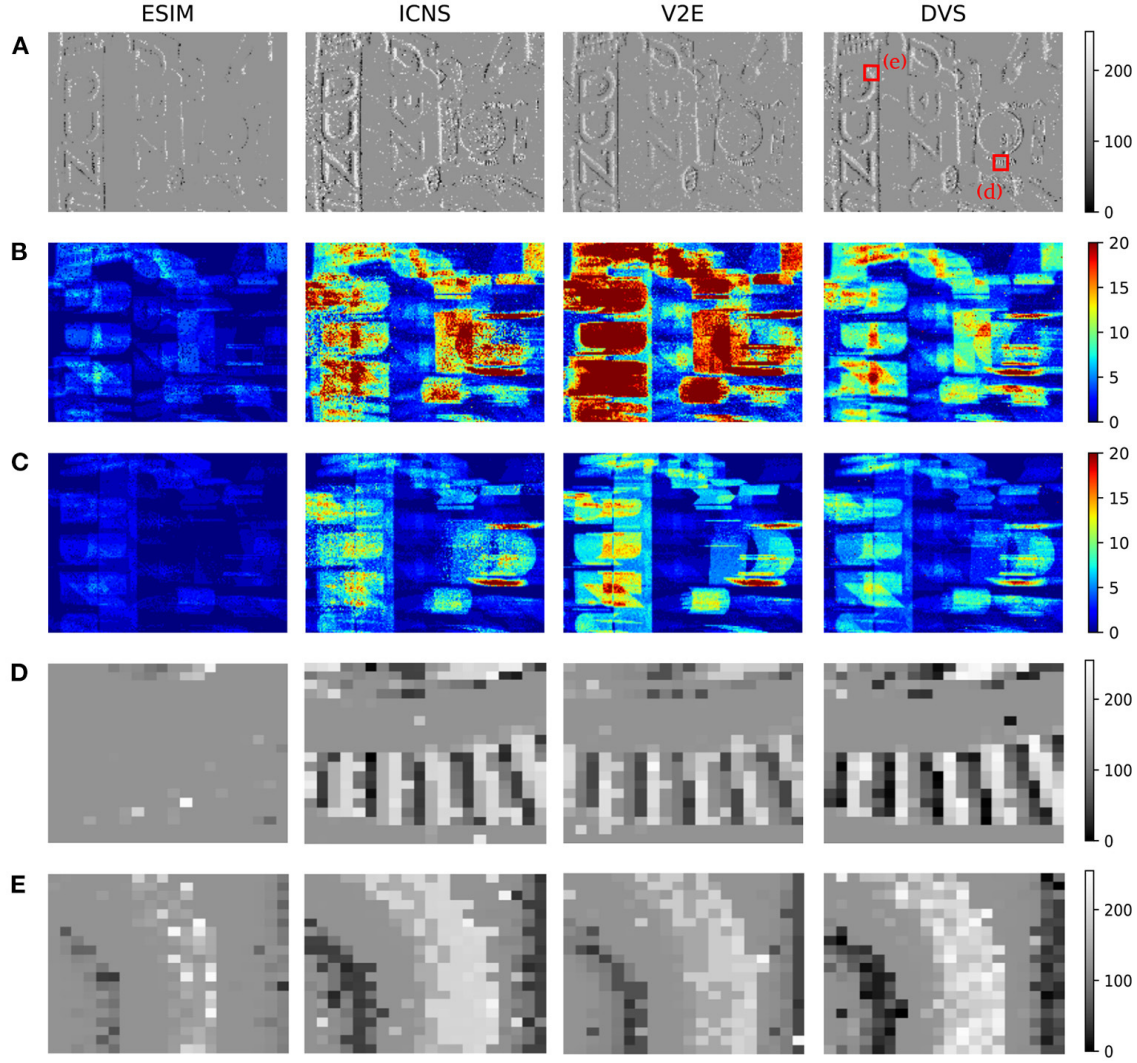
Comparison of the events generated using the different simulators with real DVS events. The input data are provided using the frames acquired with a DAVIS. **(A)** Comparison of the events integrated though a time surface (time constant 100 μ*s*). **(B,C)** Number of positive–negative events generated during the sequence. **(D,E)** Magnifications of the time surface. The column DVS corresponds to the real events generated by the DAVIS.

To benchmark the simulation model, two strategies are possible. First, the output of the models could be compared to the output of a real sensor, but in this case the scene must be precisely known to ensure that the irradiance level in the field of view of the real sensor corresponds to the one of the simulated data. As this would require precise knowledge of the properties of the optics and the scene, this work chooses to compare the data generated between the two models on complicated scenes without a perfect ground truth, and to benchmark the models compared to real data using simple characterisation experiments as illustrated in the previous section. On the other hand, Figure 8 shows the differences between the models in front of an office scene, whose light levels are acquired using the conventional pixels of a DAVIS. The two representations, the number of events and the time surface, aim at comparing both rate and temporal features of each simulator. V2E and our model are more sensitive than ESIM, which can be related to Figure 5 where these two reach the maximum probability for lower contrasts. The V2E model produces more events as its model is quicker to react, confirming the observations of Figure 4. This experiment underlines that using an office scene, the models behave slightly differently. We don't know yet if this difference significantly affects the algorithm, and further studies must correlate the quality of the simulator to the quality of the trained algorithm.

### 3.2. Revisiting the MNIST/N-MNIST Challenge

**Figure 9:**
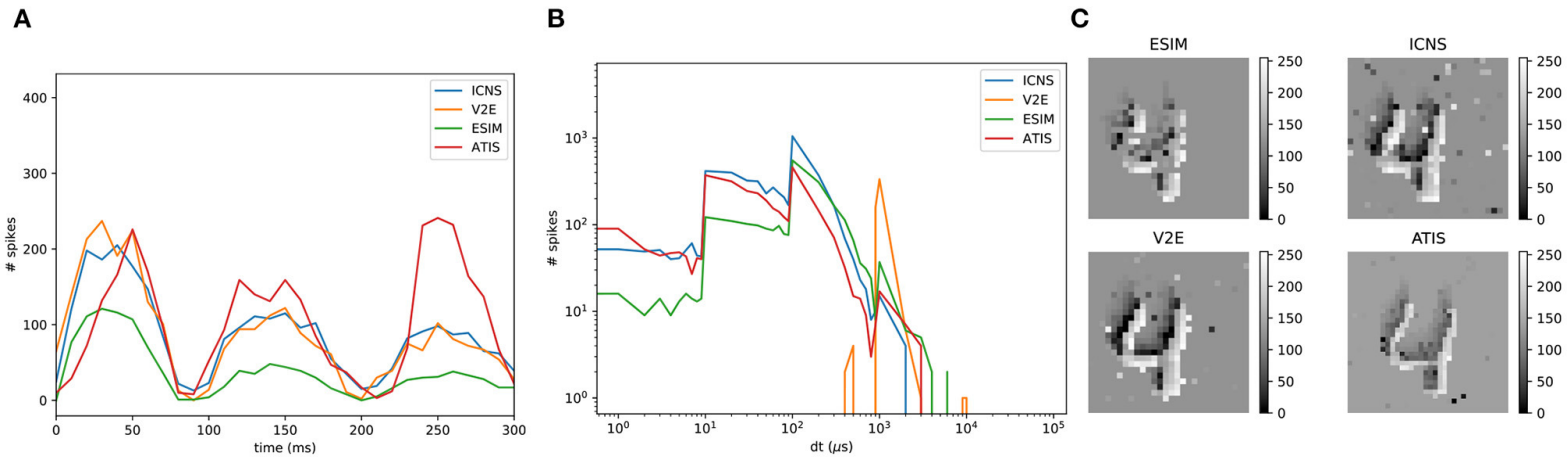
Comparison between a real DVS sensor (ATIS) and the three different simulators. **(A)** Event rate computed every 10 ms. **(B)** Histogram of the inter-spike interval. **(C)** Time surfaces.

Because DVS sensors are only sensitive to changes, the camera can be moved to produce data in a static scene. A dataset based on this idea was created by translating the sensors in front of the MNIST images (Orchard et al., 2015) by following a triangular trajectory, intended to reproduce the saccadic movement of the eye. In this experiment, the saccadic pattern lasts 300 ms, which corresponds to several frames acquired using a conventional sensor running at 50 Hz. This does not take full advantage of the temporal precision of a neuromorphic sensor over traditional frame cameras. As a purpose of illustration of our simulator, we generated a similar dataset following the same trajectory but 10 times faster: a digit is scanned during 25 ms. The digits also cross the full field of view of the sensor, and to approach the complexity of a multi-digit number, the digits are placed one after the other. To compare these two datasets, the same network is trained on the benchmark to assess its ability to handle a different environment. The architecture is structured with two convolution layers and one fully connected layer, and Spike LAYer Error Reassignment (SLAYER) (Shrestha and Orchard, 2018) was used to train the network in a supervised manner. Most of the hyper-parameters used in the network are identical to those in Shrestha and Orchard (2018), but all the time constants were reduced by a factor of 10. This accuracy achieved by the network on the original Neuromorphic MNIST (NMNIST) sequences reaches 96% on the testing set, while reaching 93% with our simulated data. Since our dataset is slightly different compared to the original NMNIST, as presented in Figure 9, it suggests that minor changes in the data can decrease performance.

## 4. Discussion

Understanding how the spikes generated in the retina are related to saccades is still an open research field. It is complicated to assess since the eye is also driven by the brain. Assuming that every digit in the MNIST database requires the same saccadic pattern to be identified is an assumption commonly made but conflicting with physiological studies (Rayner, 1998). As the motion is also constant in the simulated database, the features learnt are not robust to different speeds or trajectories: the network trained on the original NMNIST cannot be expected to work on the simulated sequences, and vice versa. The DVS pixels are triggered by motion, and thus the SNN must be resilient to diversity of dynamics in a scene. However, a reading scenario is not a dynamic problem, the movement of the eye here might rather be a consequence of our evolution and might not be intensively optimized. Artificial systems are not constrained by biological evolution constraints, and are asked to focus on one specific task. There is a chance that for this reason neuromorphic systems will inherently lag behind in terms of accuracy. The original static MNIST challenge can be extended with artificial saccades, to force the algorithm to capture the motion in order to be able to track and read the digit. Completing this new challenge would require adding kinetic energy, and thus the energy budget of the full system will encourage sensors to provide sparse data rather than redundant images.

The extensive use of simulation using inaccurate sensor models can mislead interpretations, and the models need to be compared to real data at every stage. Our work aims to make these validations as close as possible to the sensor, without requiring very precise simulations down to the transistor level. Simulation provides a much cheaper and easier exploration of new pixel designs to mimic the diversity of functions identified in the human eye. In fact, the current design of the DVS pixel does not encapsulate the diversity of features extraction performed in a biological retina. There is a need to develop more sophisticated and specialised sensors, and this is where simulations are critically important to enable the exploration of improvements to specialise and tune the DVS pixels. However, a gap still exists with the real world and such an approach could also lead to pixel designs which are impossible to manufacture. Moreover, event-based models also rely on precise simulation of the scene, which is most of the time forgotten in state-of-the-art rendering tools. Being designed for video games, they provide data without photometric units. This gap can lead to algorithms producing good results on simulated data, but inefficient in real world conditions. Imperfect simulated data can help to train a model to enhance its performance with DVS data as demonstrated in the work of Gehrig et al. (2020), but this applies for architectures neglecting the precise timing of the events. Unfortunately, deep learning models have to be trained with huge databases, which sometimes promotes quantity over quality.

## 5. Conclusion

In this work, the ESIM and V2E DVS simulators have been extended and then validated using a real sensor. The model notably improves the noise simulation by directly mapping noise distributions from real pixels. The newly proposed models of the arbiters allows to explore their impact on the data. Our model is compared against a real and fully characterized sensor using the same set of characterization experiments. This approach works to reduce the gap between simulation and real sensors beyond the conventional approach of solely comparing the quantity of events produced. It also supports novel uses of simulation to enlarge the space of neuromorphic challenges, notably to go beyond the comparison with conventional systems. Too often neuromorphic benchmarks aim to show that the same performances on similar applications can be achieved, questioning which advantages bio-inspired cameras are offering. In this regard, extending the pioneer work of the NMNIST database by adding degrees of freedom such as the sensing speed, energy budget, and sensor designs, is necessary to prove that neuromorphic systems outperform standard approaches, especially on closed-loop tasks such as reading—and not scanning—digits.

## Data Availability Statement

The datasets presented in this study can be found in online repositories. The names of the repository/repositories and accession number(s) can be found at: https://github.com/neuromorphicsystems/IEBCS.git.

## Author Contributions

DJ and GC contributed to the design of the simulator and to the experiments. DJ wrote the first draft of the manuscript. GC wrote sections of the manuscript. All authors contributed to manuscript revision, read, and approved the submitted version.

## Conflict of Interest

The authors declare that the research was conducted in the absence of any commercial or financial relationships that could be construed as a potential conflict of interest.

## Publisher's Note

All claims expressed in this article are solely those of the authors and do not necessarily represent those of their affiliated organizations, or those of the publisher, the editors and the reviewers. Any product that may be evaluated in this article, or claim that may be made by its manufacturer, is not guaranteed or endorsed by the publisher.

## Footnotes

^1^ https://github.com/neuromorphicsystems/IEBCS
